# Supplementary material for: Effect-Directed Profiling of 17 Different Fortified Plant Extracts by High-Performance Thin-Layer Chromatography Combined with Six Planar Assays and High-Resolution Mass Spectrometry
Source: Molecules. 2021 Mar 8;26(5):1468. doi: 10.3390/molecules26051468 (PMC7962818; doi:10.3390/molecules26051468)
Supplement: Supplementary file 1 [file molecules-26-01468-s001.pdf]

Supplementary Materials

Effect-Directed Profiling of 17 Different Fortified Plant Extracts by High-Performance Thin-Layer Chromatography Combined with Six Planar Assays and High-Resolution Mass Spectrometry

Gertrud E. Morlock [1,*], Julia Heil [1], Valérie Bardot [2], Loïc Lenoir [2], César Cotte [2] and Michel Dubourdeaux [2]

[1] TransMIT Center for Effect-Directed Analysis, and Chair of Food Science, Institute of Nutritional Science, Justus Liebig University Giessen, Heinrich-Buff-Ring 26–32, 35392 Giessen, Germany; Julia.Heil@ernaehrung.uni-giessen.de

[2] PiLeJe Industrie, Naturopôle Nutrition Santé, Les Tiolans, 03800 Saint-Bonnet-de-Rochefort, France; v.bardot@pileje.com, l.lenoir@pileje.com, c.cotte@pileje-industrie.com, m.dubourdeaux@pileje.com

* Correspondence: gertrud.morlock@uni-giessen.de; Tel. +49-641-9939141

Table S1 Data on the raw material and production process of the investigated 17 fortified (ipowder®) plant extracts (ID **1–4** and **6–18**) and one non-fortified extract ID **5**.

ID	Plant and/or botanical name	Dry material	Solvent	Country	Harvesting period	Drug to extract ratio	Extraction	
							Tempera–ture (°C)	Time (min)
1	Green tea (*Camellia sinensis* L. Kuntze)	final bud and two leaves	water	Ceylon	January – June 2014	2:1	70–90	30–45
2	Lemon balm (*Melissa officinalis* L.)	aerial part	water	France	Summer 2016	3:1	70–90	30–45
3	Rosemary (*Rosmarinus officinalis* L.)	leaves	water	Tunisia	Summer 2015	3:1	70–90	30–45
4	*Eleutherococcus senticosus* [Rupr. et Maxim.] Maxim	roots	water	China	Autumn 2016	3:1	60–80	45–60
5	Green tea (*Camellia sinensis* L. Kuntze)	final bud and two leaves[a]	NA	Ceylon	January – June 2014	–	–	–
6	Yerba mate (*Ilex paraguariensis* A. St.–Hil.)	leaves	water	Brazil	Summer 2017	3:1	70–90	30–45
7	Red vine (*Vitis vinifera* L.)	leaves	water	Tunisia	Autumn 2014	3:1	70–90	30–45
8	Valerian (*Valeriana officinalis* L.)	roots	water	Poland	Winter 2016	2:1	60–80	45–60
9	Meadowsweet (*Spiraea ulmaria* L.)	floral tops	water	Poland	Summer 2016	3:1	70–90	30–45
10	*Echinacea purpurea* (L.) Moench	roots	water	France	Autumn 2015	2:1	60–80	45–60
11	Black currant (*Ribes nigrum* L.)	leaves	water	France	Summer 2016	3:1	70–90	30–45
12	Black radish (*Raphanus sativus* var. niger (Mill.) J.Kern.)	roots	water	France	Winter 2015	3:1	60–80	45–60
13	Horse tail (*Equisetum arvense* L.)	aerial part	water	Bulgaria	Summer 2017	2:1	70–90	30–45
14	Hops (*Humulus lupulus* L.)	cones	water	Poland	Autumn 2017	2:1	70–90	30–45
15	Grape (*Vitis vinifera* L.)	pomace[b]	ethanol – water 3:7	France	Autumn 2012	5:1	30–50	45–60
16	Passiflora (*Passiflora incarnata* L.)	aerial part	water	France	Summer 2017	2:1	70–90	30–45
17	Artichoke (*Cynara scolumus* L.)	leaves	water	France	Summer 2015	2:1	70–90	30–45
18	*Eschscholzia californica* Cham.	aerial part	water	France	Summer 2017	2:1	70–90	30–45

[a]Same raw material as that used to make the fortified extract of green tea (ID 1).
[b]Grape pomace was fresh unlike the other plants, which were dry.

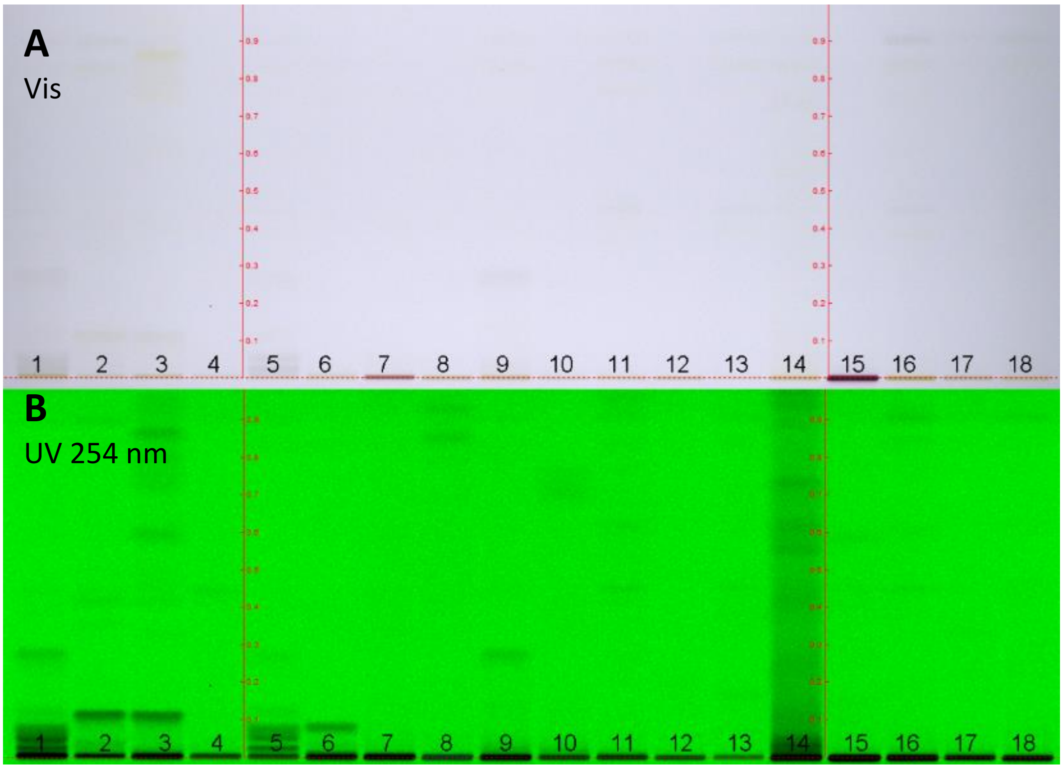

Figure S1. HPTLC chromatograms of the 18 investigated plant extracts (track assignment in Table S1; 200 µg applied each) developed with the apolar mobile phase MP 2 and recorded at white light illumination (A, visible, Vis) and UV 254 nm (B); this plate was subjected to the *A. fischeri* bioassay (bioautogram in Figure 2A).

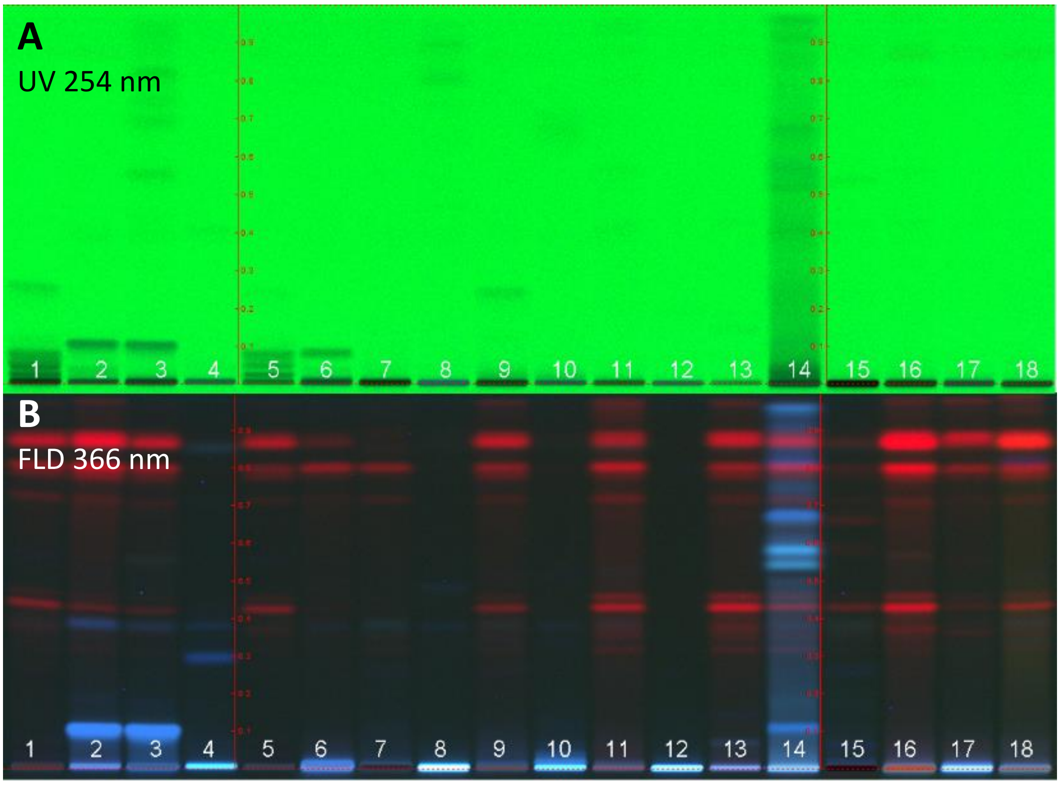

Figure S2 HPTLC chromatograms of the 18 investigated plant extracts (track assignment in Table S1; 200 µg applied each) developed with the apolar mobile phase MP 2 and recorded at UV 254 nm (A) and FLD 366 nm (B); this plate was subjected to the *α*–glucosidase inhibition assay (autogram in Fig. 2B).

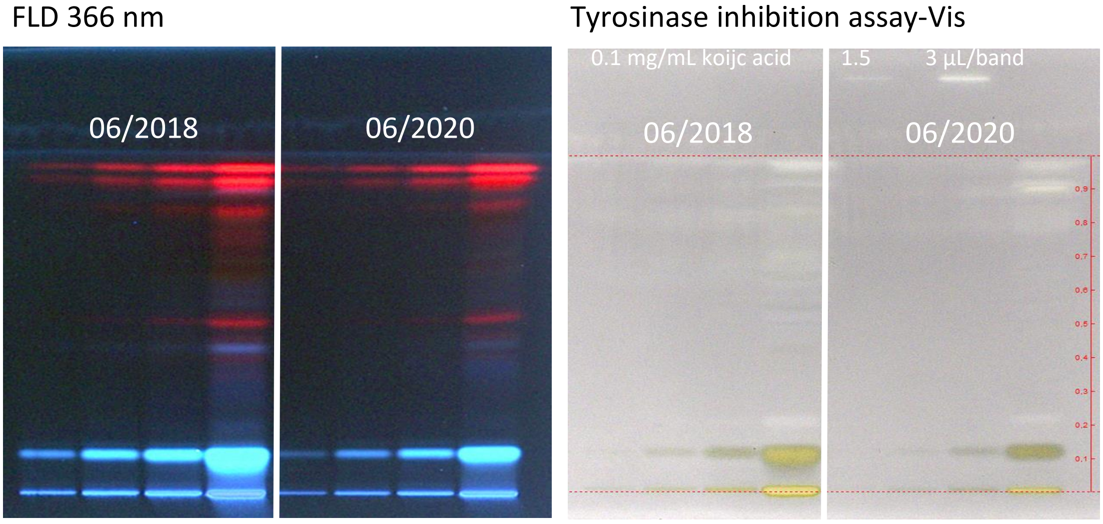

Figure S3. Stability check after 2 years, exemplarily shown for the fortified plant extract of rosemary ID **3** (Table S1; 20, 50, 100 and 400 µg applied each) via HPTLC chromatograms at FLD 366 nm developed with the apolar mobile phase MP 2, and HPTLC tyrosinase inhibition autograms at white light illumination (visible, Vis).
